# Pivot Step Jump: A New Test for Evaluating Jumping Ability in Young Basketball Players

**DOI:** 10.3390/jfmk7040116

**Published:** 2022-12-15

**Authors:** Apostolos S. Theodorou, Hariklia-Parthenia Rizou, Emmanouil Zacharakis, Ioannis Ktistakis, Evangelos Bekris, Vassilios Panoutsakopoulos, Panagiotis Strouzas, Dimitrios I. Bourdas, Nikolaos Kostopoulos

**Affiliations:** 1 School of Physical Education & Sport Sciences (TEFAA), National and Kapodistrian University of Athens, Dafni, 17237 Athens, Greece; 2 Biomechanics Laboratory, School of Physical Education and Sports Sciences at Thessaloniki, Aristotle University of Thessaloniki, 54124 Thessaloniki, Greece

**Keywords:** vertical jump, interlimb asymmetry, performance, power, sport-specific skill, developmental age, motor skill assessment

## Abstract

Jumping ability in basketball is usually assessed using standardized vertical jump tests. However, they lack specificity and do not consider the player’s basketball skills. Several studies have suggested performing specific jump tests, which are tailored to the movement patterns and requirements of a basketball game. The pivot step jump test (PSJT) is a novel test designed to evaluate the specific jumping abilities of basketball players by combining a pivot step on one leg with a maximum bilateral vertical jump. This study had two aims: to determine the reliability and validity of the PSJT using typical jump tests as the criterion measure and to demonstrate the PSJT as a practical test to evaluate specific jumping ability in young male and female basketball players. Twenty female (EGA; 14.0 ± 0.7 years, 59.3 ± 7.9 kg, 162.1 ± 5.5 cm) and fifteen male (EGB; 14.0 ± 0.7 years, 58.1 ± 7.7 kg, 170.3 ± 6.4 cm) basketball players participated in the study. The test–retest reliability of the PSJT within sessions (intrasession reliability) and across sessions (intersession reliability) was assessed within EGA. For the evaluation of validity, EGB performed the PSJT and a series of criterion jumping tests. For EGA, no changes (*p* > 0.05) were found in PSJT performance between test sessions and excellent intra- and intersession reliability was observed (ICCs > 0.75). Correlation coefficients indicated high factorial validity between the jumping tests and PSJT (*r* = 0.71–0.91, *p* < 0.001). The PSJT appears to offer a valid assessment of jumping ability in basketball and is a practical test for assessing sport-specific jumping skills in young basketball players.

## 1. Introduction

Basketball is a physically demanding sport where success dependents on a variety of fundamental physical skills such as acceleration, quickness, strength, and power [1,2]. During a basketball game, bilateral and unilateral jumps are performed at the frontal and sagittal plane of motion. However, most training programs emphasize drills, which are performed at the sagittal plane and rarely examine the effects of training at the other planes of motion. The specialization of training implies that fitness assessment should include actions that are kinematically similar to the movements of a given sport. Jumping is one of the basic actions performed during a basketball game, as the basket is at a height of 3.05 m [3]. Jumping ability is a manifestation of power, which is a sport-specific feature of basketball [4]. All actions involving jumps are affected by a range of different factors pertinent to the game of basketball [2]. A large variety of the offensive and defensive skills used in the sport, such as shots, lay ups, rebounds, etc., involve jumping. Jumping ability, therefore, determines the performance and level of basketball players [2] and should be adapted to the specific requirements the game.

Jumping ability in basketball is widely assessed with a wide range of standardized jumping tests [5], i.e., the squat jump, countermovement jumps with or without arm swing, the drop jump, and the standing long jump [3,6,7]. Performance in these tests assesses the players’ ability to utilize the stretch shortening cycle and/or arm swing to increase jump height [8]. In the case of the countermovement jump, its execution with (CMJA) or without arm swing (CMJ) is suggested to be reliable and valid in measuring peak power of lower limbs and jump height [9,10]. Furthermore, the difference between CMJ and CMJA provides an indication of neuromuscular ability to coordinate intra-segmental energy flow to achieve higher power and jump height [11,12,13]. However, basketball players differ from other athletes in terms of both the magnitude of power output and the time instance of its peak, as they execute the vertical jumps in a sport-specific force- and time-dependent pattern [14,15,16,17,18,19,20]. It can be argued that typical vertical jump tests are generic regarding the performance evaluation of basketball players since they lack specificity and do not consider the player’s basketball skills. For example, in basketball, players perform a variety of different types of jumps, i.e., jumps from a still position and jumps following a running action [21]. These jumps can be influenced by different factors related to the game, such as the path of the ball, physical contact with opponents and the phase of the game, offense, or defense [22]. Several studies have questioned the validity of these tests in assessing the functional abilities of basketball players and have suggested that specific jump tests tailored to movement patterns and the demands of a basketball game be performed [22,23]. In addition, it has been suggested that performing vertical jump tests on the court is more appropriate and appealing for basketball players when evaluating their jumping ability [10]. In addition, basketball-specific physical field tests are important for monitoring training effectiveness and fitness status [24]. In the case of jumping, the specific jump tests used in basketball are the “three-steps approach with two leg take-off vertical jump” test, the “two-steps approach with one leg take-off vertical jump” test [2], and the “one-step jump” test [25]. The concept underlying the aforementioned jump tests is to assess specific aspects of basketball players’ strength and conditioning abilities by combining jumping with game-specific skills that involve a jumping action.

To be effective in the game and utilize his jumping ability, a player must adapt this ability to the context and specific requirements of the sport. Physical qualities and tactics are currently considered as two inseparable representations of a player’s actions, and therefore it is crucial to take a more ecological approach when training and evaluating athletes [26]. For basketball players to be successful, they must be able to carry out multiple power-based actions before jumping such as cutting and dribbling simultaneously. Therefore, measuring vertically oriented power-related attributes requires a targeted approach that the current power-related tests perform with limited ecological validity. The present study proposes a novel jump test for a functional assessment of basketball jumping abilities. Pivoting is when a player stands still and steps with one foot. The foot that stays on the ground is called the pivot foot. A player that has the ball and is standing still may step with one foot in order to change direction and pass the ball or to avoid opponents and take a shot. Pivots and jumps are combined in all these actions. The pivot step jump is a step on one leg (while the other remains in contact with the ground) for changing direction, followed by a maximum vertical jump on both feet. Basketball players usually perform this movement after rebounds and to find a better position for a shot when blocked by the opponents [3]. The jump following a pivot has never been studied and evaluated as a test. Considering that talent identification and long-term development require the inclusion of tests of technical ability and tactical behavior [27], the purpose of this study was to investigate the validity and reliability of a new basketball jump test, that involves a vertical jump following a pivot action. The test was referred to as the pivot step jump test (PSJT). We hypothesized that the PSJT would have (a) high intrasession, intersession and interrater reliability and (b) a strong relation with the vertical jump tests most used to evaluate power in basketball players.

## 2. Materials and Methods

### 2.1. Experimental Approach to the Problem

The present study was designed to determine the concurrent reliability and validity of a new jump test by examining correlations with established and previously validated jump tests. Concurrent validity is a type of criterion-related validity in which correlation coefficients are calculated between a true criterion and an alternative measure. Previous research has proposed using this method to assess the validity of upper-body power tests [28] and lower-body power [29]. To test the reliability and validity of the proposed jump test, each participant performed the test in four instances. To avoid a possible training effect due to the athletes’ prolonged and repeated participation in tests with maximal effort, two groups of participants were tested, with each group assigned to test each hypothesis.

### 2.2. Participants

Fifteen male (EGA; age: 14.0 ± 0.65 years) and twenty female basketball players (EGB, aged 14.0 ± 0.65 years) participated at the study. The anthropometric characteristics of the participants are shown in Table 1. All athletes had at least 5 years of experience in basketball and competed in the first division of their age group. All experimental procedures were approved by the Institutional Research Ethics and Bioethics Committee (1063/13 June 2018). All participants and their guardians were informed about the benefits and risks of the study. Signed parental consent was obtained for the participation of minor athletes.

### 2.3. Experimental Procedure

**Figure 1:**
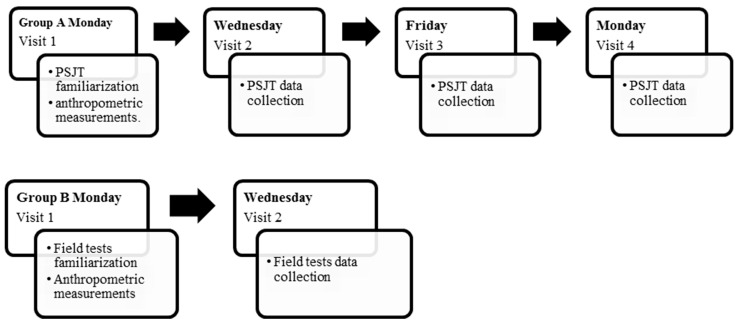
Graphical representation of the design of the study for group A (EGA) and group B (EGB).

All testing was conducted in the off-season to avoid the effects of team training. EGA group was used to determine test–retest reliability of the new jump test within sessions (intrasession reliability), within investigators (interrater reliability) and across sessions (intersession reliability). EGB group was used to determine the validity of the new test with four traditional jump tests. Participants at the EGA group reported to the laboratory on four separate occasions (Figure 1).

On the first visit, participants familiarized themselves with the PSJT and took anthropometric measurements (mass, standing height, seated height, leg length, and shin length). Measurements were performed according to Carter and Heath [30], using the Seca 220 telescopic measuring rod, the Seca Alpha 770 scale and Seca 201 measurement tape (Seca GmbH & Co., Hamburg, Germany). The anthropometric data were used to estimate the maturity level of the participants by using the simplified regression equations for maturity adjustment proposed by Moore et al. [31].

Prior to testing, all participants completed an 8-min warm-up consisting of submaximal plyometric and jump drills. During the familiarization period, detailed instructions were provided on how to execute the PSJT and 4 trials were performed with emphasis on proper execution technique.

**Figure 2:**
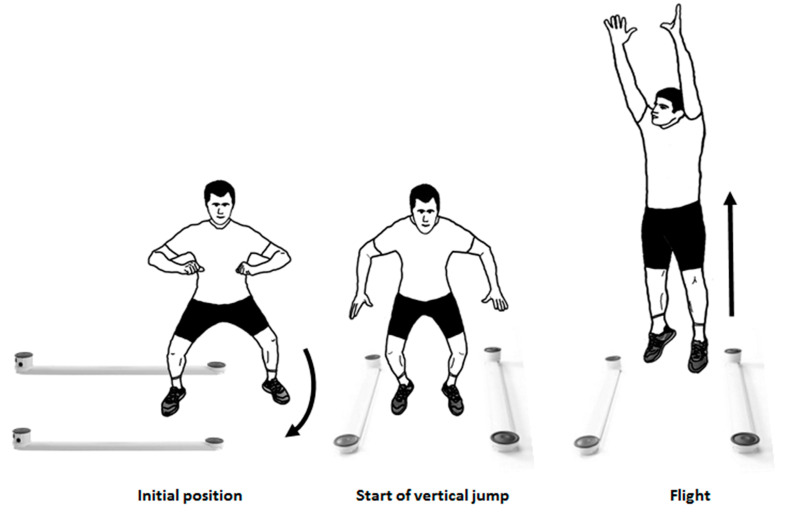
Graphical representation of the design of the study.

The PSJT was administered using the Optogait system (Microgate, Bolzano, Italy). Participants commenced from a stationary semi-squat position (knee angle of 90°), with one leg (right, PSJT_RLEG_ or left, PSJT_LLEG_) inside the Optogait’s measuring rods. Then, they performed a forward 90° pivot step followed by a rapid vertical jump, using an arm swing aiming to reach maximum height. During the PSJT, participants were allowed to perform a countermovement of the legs and arms (Figure 2).

The height of the jump was recorded as the result of the test. Three attempts were allowed for each leg. The mean of the two best attempts was used for analysis. The same procedure was then repeated for the other leg. Following a two-day rest period (Visit 2), participants performed the PSJT twice (tests 1 and 2), separated by a 30 min interval, under the supervision of investigator “A”. A third PSJT was performed two days later (Visit 3), under the supervision of investigator “B”, and a fourth PSJT was performed seven days later (Visit 4), again under the supervision of investigator “B”.

EGB participants reported to the laboratory on two separate occasions to familiarize themselves with the testing procedure and data collection. On the first visit, participants completed a familiarization with the PSJT and the four jump tests and took anthropometric measurements. After two days (Visit 2), participants repeated the five jump tests. The countermovement jump (CMJ) commenced from a stationary upright standing position (hands akimbo) followed by a preliminary downward movement by flexing the knees and hips and subsequently vigorously extending them to perform a vertical jump [32]. The countermovement jump with arm swing (CMJA) was conducted following the same procedure as the CMJ, but participants were allowed to perform the jump with an arm swing. The effect of arm swing on countermovement was determined using the arm swing augmentation index (AS_INDEX_), which was calculated as the percentage ratio of the difference in jump height between the CMJA and CMJ divided by the jump height in CMJ. The countermovement jumps with the right or left leg (CMJ_RLEG_ and CMJ_LLEG_) were performed similarly to the CMJA, but with one leg. Performance in all jump tests was evaluated by jump height, estimated from the time of flight measured by the Optogait system [22,33]. To investigate the presence of possible asymmetry between CMJ_RLEG_ and CMJ_LLEG_, and between PSJT_RLEG_ and PSJT_LLEG_, the respective asymmetry values were quantified based on the symmetry angle (θ_SYM_) [34].

An additional jump test to assess lower extremities explosive strength, was the standing long jump (SLJ). The SLJ commenced with the toes behind a take-off line. By bending the knees and swinging the arms freely, the participant performed a horizontal jump to cover the greatest horizontal distance possible. The distance was measured from the take-off line to the rearmost heel [35]. The jump tests were performed in a random order. For each test, three attempts were allowed with a 1-min rest period between attempts and 8 min between tests. The mean of the two best attempts was used for analysis.

### 2.4. Statistical Analysis

Statistical analysis for this study was performed using IBM SPSS Statistics v.27.0.1.0 (IBM Corp., Armonk, NY, USA) software. The level of significance was set at *a* = 0.05. After assessing the normality of the data (each test session for each leg) with the Kolmogorov–Smirnov test, the means and standard deviations for all variables were calculated. The *p* values obtained by the Kolmogorov–Smirnov analyses were all above 0.05, indicating that the data were normally distributed. The intrasession (test 1 vs. test 2), interrater (test 2 vs. test 3), and intersession (test 3 vs. test 4) reliability of the PSJT measures was quantitatively assessed with two-way random, single measure intraclass correlation coefficient (ICC) and their respective 95% CI. An ICC with values >0.75, ≥0.40 and ≤0.75, and <0.40 indicated “excellent”, “fair to good”, and “poor” reliability, respectively [36]. In addition, Bland–Altman plots were used to determine the extent of agreement between test–retest values. The difference in the paired intrasession, interrater and intersession measures was plotted against their respective means. The evaluation criterion was that 95% of the data points should lie within the mean ± 2 SDs of the differences for the intra and intersession measurements, which corresponds to the 95% CI. Absolute reliability was calculated using the standard error of measurement (SEM) [37], which was then expressed as a percentage of the mean value with the coefficient of variation (CoV). A CoV value less than 10% was set as a criterion for an acceptable reliability [22]. The minimal difference (MD) in absolute terms (cm) and as a percentage of the mean value (MD%) was also determined [37]. The factorial validity of the jump tests and the relationships between the measured variables were determined using Pearson’s correlation coefficients.

## 3. Results

The seated height and the lengths of the body segments measured are shown in Table 2. The maturity offset was found to be positive in both groups.

### 3.1. PSJT Reliability

Descriptive statistics of the testing sessions for EGA are provided in Table 3. The calculated ICC and 95% CI values for the PSJT performances across the three reliability analyses are presented in Table 4. The ICCs were all above 0.75, indicating an excellent intra and intersession reliability.

**Figure 3:**
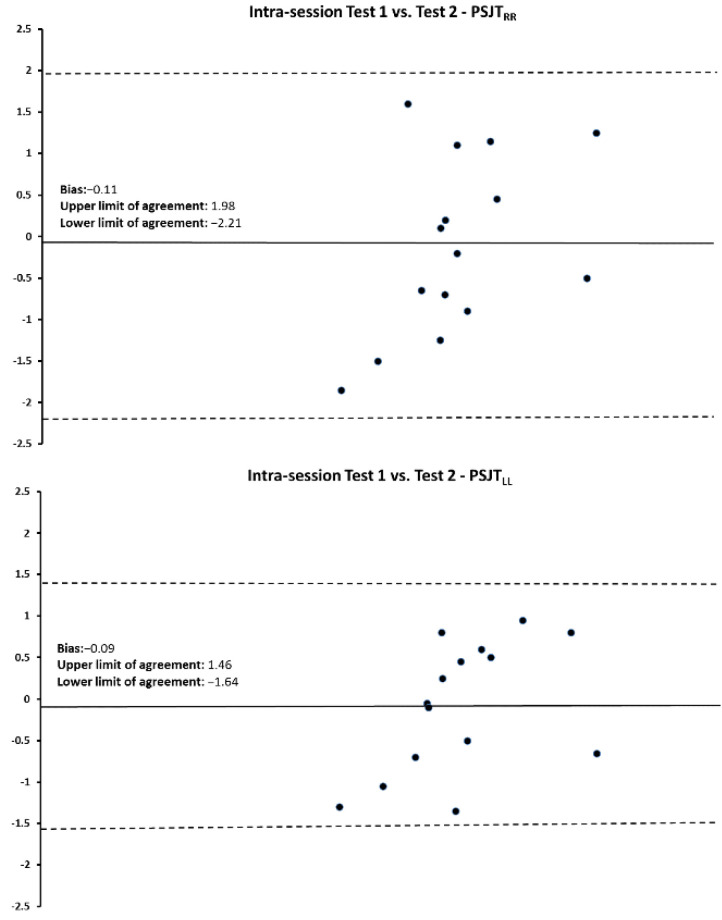
Bland–Altman plot for the intrasession reliability between PSJT_RLEG_ and PSJT_LLEG_ for EGA. The difference between the two measurements per subject is plotted against the mean of the two measurements. The dotted lines represent the upper and lower limits of agreement for 95% CI.

**Figure 4:**
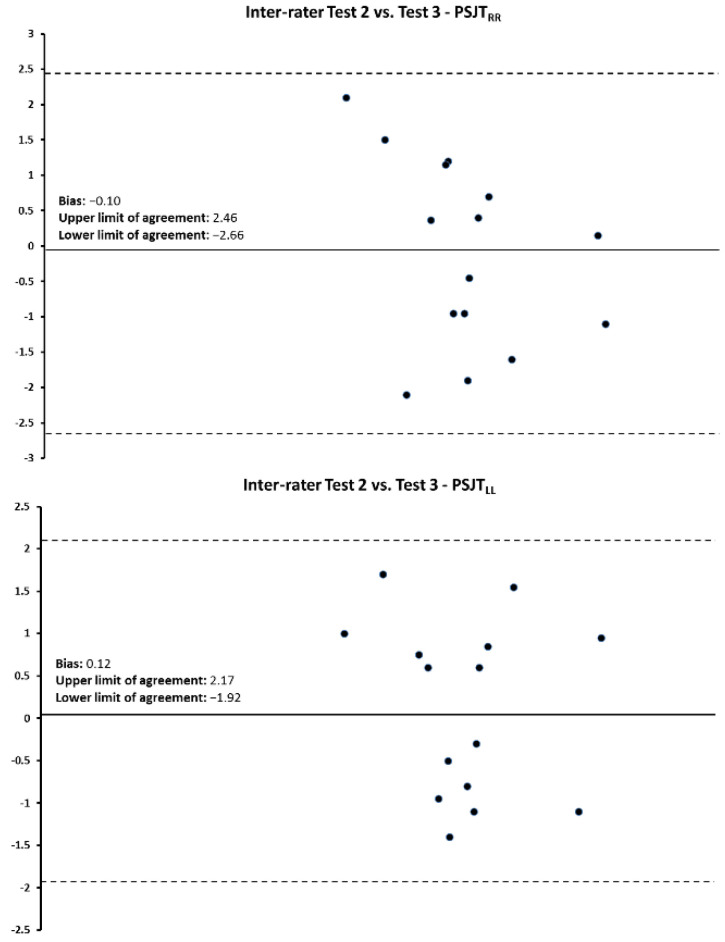
Bland–Altman plot for the interrater reliability between PSJT_RLEG_ and PSJT_LLEG_ for EGA. The difference between the two measurements per subject is plotted against the mean of the two measurements. Dotted lines represent the upper and lower limits of agreement for 95% CI.

**Figure 5:**
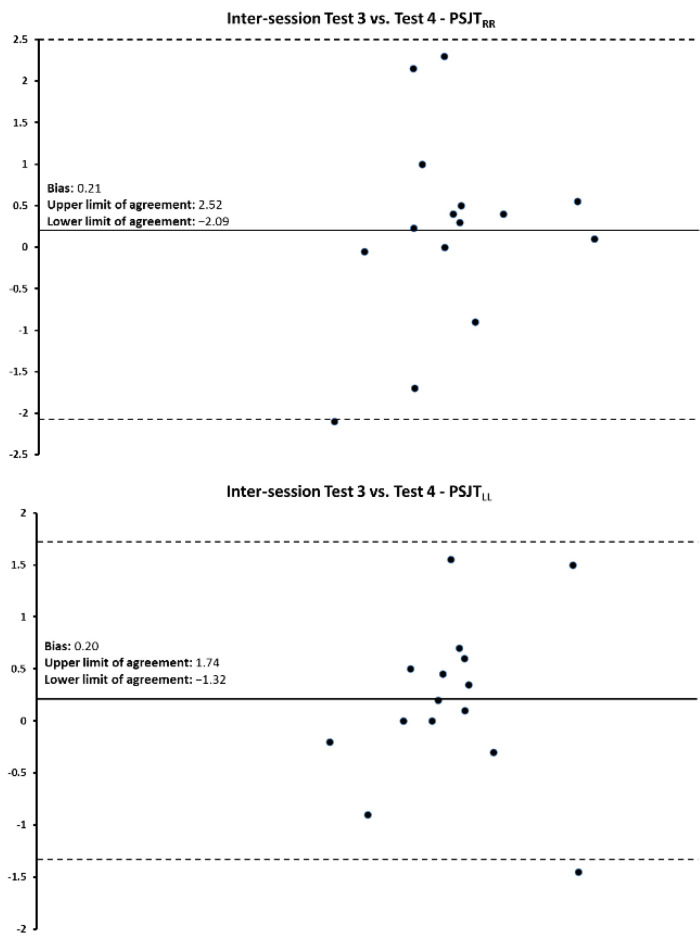
Bland–Altman plot for the intersession reliability between PSJT_RLEG_ and PSJT_LLEG_ for EGA. The difference between the two measurements per subject is plotted against the mean of the two measurements. The dotted lines represent the upper and lower limits of agreement for 95% CI.

Bland–Altman plots and the regression analyses results for the PSJT performances for the intra- and inter-session comparisons are shown in Figure 3, Figure 4 and Figure 5 and Table 5. For all comparisons, data points were within the mean ± 2 SD.

### 3.2. PSJT Validity

Table 6 shows the results of all jumping tests examined. No significant interlimb asymmetry was observed in both the CMJ and the PSJT.

All correlation coefficients indicated significant correlations among the jump tests (*p* < 0.05). When univariate associations between variables were examined, correlations between jump tests and PSJT performances ranged from 0.71 to 0.91, indicating that the jump tests assessed and the PSJT have high factorial validity (Table 7).

## 4. Discussion

The purpose of this study was to investigate the reliability of the basketball-specific assessment of jumping ability, the PSJT and its validity with the jump tests commonly used to assess explosive strength and power in basketball players. Overall, the results of this study showed excellent intersession and intrasession reliability for the PSJT in young male basketball players and validity with standard jump tests in young female basketball players.

There are few studies in the literature on specific jump tests tailored to the kinematic and technical demands of a basketball game [22]. Previous studies have reported the reliability of CMJ, squat jump and drop jump tests in basketball players [25,38]. Although different methodological approaches were used, reported reliability ranged from very good to high, with CV values of 3 to 4% [25], Cronbach’s alpha > 0.90 [38], and test–retest correlation of 0.98 [39]. Markovic et al. [40] also reported similar values for CMJ reliability (ICC 0.96) for one-session reliability (intrasession), while Moir et al. [41] reported equally high ICC reliability values (0.87–0.95) and CV (4.0–6.6%) for the same test between multiple sessions (intersession). The low CV values (between 1.3% and 2.4%) reported for the PSJT in the presented study suggest high reliability as well as low variation in performance between the first and subsequent trials. This also suggests an ease of administration of the test and a low motor learning effect in male basketball players.

Our finding of excellent intra- and intersession reliability was based on the mean of four consecutive trials each acquired with the mean of the two best attempts in each leg, which is consisted with previous studies [42,43]. An interesting observation is that when the test was performed with the right leg the PSJT tended to have slightly lower ICC values and higher SEM, CV, and MD values.

In the present study, all correlations between the PSJT and jump tests were strong, suggesting that the PSJT is a concurrently valid test for assessing jumping ability in basketball. This confirms past research that has demonstrated that the standing long jump and the CMJ test are the most reliable jump tests for assessing the explosive properties of the lower limbs in physically active athletes [22,40,44].

The CMJ is thought to provide an assessment of the ability to generate force rapidly during stretch-shortening cycle movements [45]. Two types are commonly used when performing the CMJ. The first involves the use of the arm swing (CMJA), while the second limits the influence of the arm swing by requiring the athlete to keep their hands on the hips [3,46]. Previous research found that the jump height is the same between CMJ and the jump shot [21]. However, in the case of PSJT, the 90-degree pivot step is followed by a fast vertical jump with a countermovement of the legs and the swinging of the arms. The apparent similarity between PSJT and CMJA is the countermovement of the legs and arms during the pivot action. When pivoting from an upright standing position, basketball players must also perform a preparatory downward movement by flexing the knees and hips before performing a rapid vertical jump. Heishman et al. [47] have shown that both CMJA and CMJ provide valid information for assessing jumping ability, but each offers distinct advantages. CMJ is useful for assessing performance changes on a long-term basis, such as changes in performance across training periods [21]. However, several authors suggest that the inclusion of an arm swing, when performing a CMJ test, leads to a higher degree of sport specificity that may improve reliability [48,49]. In the present study, the AS_INDEX_ was within the range of values reported in the past for young and adult basketball players, suggesting that the participants’ intersegmental neuromuscular coordination pattern among was of a good standard [8].

From a biomechanical perspective, vertical jumps with arm swing increase jump height due to increased power and work output [8,11,50]. Specifically, the arm swing generates work in the shoulder joint and the flow of this work leads to an increase in torque in the hip joint, ultimately resulting in a higher jump height [11,12,51,52]. This mechanism may provide a basis for the finding that jump heights were higher in the PSJT tests than in the corresponding CMJ tests. In addition, previous research has shown that the effect of arm swing remains unchanged during developmental age in young basketball players [8]. Therefore, it can be hypothesized that the PSJT may also be a sport-specific jump test in adult basketball players and that future research should address this issue.

Despite the fact that the arm swing is involved in all jumping actions in basketball, previous research suggests that the CMJ had marginally larger reliability scores than the CMJA [53]. This may be due to the significant interlimb asymmetry in leg stiffness observed in basketball players performing the CMJA [54]. As leg stiffness is an important factor in generating power during jump tests [55], interlimb asymmetry in lower extremity power output results in decreased jump performance in athletes [56,57]. However, in the present study, the strong correlations when both the right and left leg were used to perform the PSJT suggest that countermovement was not affected by limb or side preference. This was also confirmed by the extracted θ_SYM_ values, where θ_SYM_ is recommended for determining differences between limbs [58]. Further confirmation of this observation was the strong and significant correlation between CMJ_RLEG_ (*r* = 0.77, *p* < 0.001) and CMJ_LLEG_ (*r* = 0.83, *p* < 0.001) with PSJT. This is consistent with the literature, as previous studies found high reliability scores for the unilateral CMJ in basketball players [59]. In addition, the performance of the PSJT with a 90° pivoting step may have favored the absence of interlimb asymmetry, as young basketball players showed a large intralimb asymmetry during a 180° change of direction test [60], with a low to moderate association with bilateral CMJ deficit in male and female players [61].

A high correlation was also found between the PSJT and the SLJ. The standing long jump is considered an indicator of maximal horizontal power generation in the sagittal plane [62]. Due to its correlation with basketball performance variables, its use to assess lower-body power in athletes is considered appropriate [44]. According to Wen et al. [62], players with high scores in SLJ are likely to be efficient in performing explosive short burst actions on the court. Pivoting with a subsequent jump shot is also a quick, powerful action that helps players gain an advantage over their opponents by rapidly moving into a position where they can score easily. Therefore, the high correlation with the SLJ likely indicates its suitability as a method for assessing sport-specific, power-related attributes in basketball.

Longitudinal examination of between-limb differences has been shown to be important for long-term monitoring of sport performance, as well as the accuracy of interpretation of asymmetry scores in bilateral and unilateral tests [63]. The lack of such monitoring is a limitation to the study. Although the literature does not support a gender bias in jump kinetics in young basketball players [64], the correlation of the PSJT with standard vertical jump tests should also be investigated and confirmed in young basketball players. In addition, the possible effect of the playing position [65] and level [66] that is evident in young basketball players was not considered. Despite the existence of contradicting findings in past research concerning the effect of the playing position on jumping performance [3], future research should consider the positional and playing level differences in the PSJT as well.

## 5. Conclusions

In summary, the results of this study suggest that the PSJT is a concurrent valid measure of jumping ability for estimating lower limb explosive force in young female basketball players. Its strong correlation with vertical jump tests, which are commonly used to assess jumping ability and explosive power in basketball players, makes it useful for monitoring changes in jumping performance. In addition, the movement pattern tailored to the demands of a basketball game provides coaches with an ecologically valid and reliable test for quantifying adaptation to training programs.

## Figures and Tables

**Table 1:** Anthropometric characteristics of the participants (mean ± SD).

Group	Body Height (cm)	Body Mass (kg)	Body Mass Index (kg/m^2^)
EGA (males; *n* = 15)	170.26 ± 6.43	58.13 ± 7.69	20.05 ± 2.89
EGB (females; *n* = 20)	162.07 ± 5.48	59.29 ± 7.87	22.57 ± 2.63

EGA: Experimental group A; EGB: Experimental group B.

**Table 2:** Mean ± SD of the anthropometric measurements (*n* = 15).

Group	Seated Height (cm)	Leg Length (cm)	Shin Length (cm)	Maturity Offset (yrs)
EGA (males; *n* = 15)	87.57 ± 3.47	110.14 ± 4.21	45.46 ± 2.82	0.9 ± 0.4
EGB (females; *n* = 20)	84.95 ± 3.51	102.42 ± 4.05	41.67 ± 1.65	2.7 ± 0.3

EGA: experimental group A; EGB: experimental group B.

**Table 3:** Mean ± SD of the PSJT measures obtained during all testing sessions for EGA (*n* = 15).

Test	PSJT_LLEG_ (cm)	PSJT_RLEG_ (cm)
Test 1	37.31 ± 6.09	36.97 ± 6.24
Test 2	37.40 ± 5.71	37.02 ± 5.82
Test 3	37.27 ± 5.90	37.12 ± 6.32
Test 4	37.07 ± 5.82	36.91 ± 6.05

PSJT_LLEG_: pivot step jump test on the left leg; PSJT_RLEG_: pivot step jump test on the right leg.

**Table 4:** Interclass correlation coefficients (ICC), 95% confidence interval (95% CI), standard error of measurement (SEM), coefficient of variation (CoV), and minimal difference (MD) values of the intrasession, interrater, and intersession reliability analysis of the PSJT measures for EGA (*n* = 15).

Reliability Analysis	Intra-Session		Inter-Rater		Inter-Session	
	PSJT_LLEG_	PSJT_RLEG_	PSJT_LLEG_	PSJT_RLEG_	PSJT_LLEG_	PSJT_RLEG_
ICC	0.992 *	0.981 *	0.98 5 *	0.978 *	0.991 *	0.983 *
95% CI	0.975–0.997	0.946–0.994	0.956–0.995	0.970–0.993	0.975–0.987	0.950–0.994
SEM (cm)	0.694	0.810	0.694	0.876	0.544	0.786
CoV (%)	1.384	2.190	1.859	2.364	1.463	2.124
MD (cm)	2.541	3.981	3.41	4.307	2.673	3.863
MD (%)	6.803	10.759	9.135	11.618	7.191	10.438

*: *p* < 0.01; PSJTL_LEG_: pivot step jump test on the left leg; PSJT_RLEG_: pivot step jump test on the right leg.

**Table 5:** Interclass correlation coefficients (ICC), 95% confidence interval (95% CI), standard error of measurement (SEM), coefficient of variation (CoV), and minimal difference (MD) values of the intrasession, interrater and intersession reliability analysis of the PSJT measures for EGA (*n* = 15).

Comparisons	*F*	*p*	*R*^2^ Adjusted
Intrasession Test1 vs. Test2—PSJT_LLEG_	(1,13) = 926.05	<0.001	0.986
Intrasession Test1 vs. Test2—PSJT_RLEG_	(1,13) = 360.06	<0.001	0.963
Interrater Test2 vs. Test3—PSJT_LLEG_	(1,13) = 406.73	<0.001	0.967
Interrater Test2 vs. Test3—PSJT_RLEG_	(1,13) = 319.17	<0.001	0.958
Intersession Test3 vs. Test4—PSJT_LLEG_	(1,13) = 731.99	<0.001	0.981
Intersession Test3 vs. Test4—PSJT_RLEG_	(1,13) = 370.75	<0.001	0.964

**Table 6:** Descriptive statistics for all tests and indexes examined in the present study for EGB (females, *n* = 20).

Test	Mean ± SD	95% CI
CMJ (cm)	23.04 ± 4.08	21.13–24.95
CMJA (cm)	26.64 ± 4.61	24.48–28.80
AS_INDEX_ (%)	15.91 ± 7.24	12.51–19.29
CMJ_RLEG_ (cm)	11.48 ± 2.45	10.33–12.62
CMJ_LLEG_ (cm)	11.00 ± 2.39	9.87–12.12
θ_SYM-CMJ_ (deg)	−1.46 ± 4.53	−3.57–0.66
PSJT_RLEG_ (cm)	25.46 ± 4.42	23.39–27.53
PSJT_LLEG_ (cm)	25.22 ± 4.65	23.04–27.40
θ_SYM-PSJT_ (deg)	−0.35 ± 2.40	−1.47–0.77
SLJ (cm)	155.50 ± 25.47	143.57–167.42

CMJ: bilateral countermovement jump—arms akimbo; CMJA: bilateral countermovement jump with an arm swing; AS_INDEX_: arm swing augmentation index; CMJ_RLEG_: unilateral countermovement jump performed with the right leg; CMJ_LLEG_: unilateral countermovement jump performed with the left leg; θ_SYM-CMJ_: symmetry angle for the unilateral countermovement jumps; PSJT_RLEG_: pivot step jump performed with the right leg; PSJT_LLEG_: pivot step jump performed with the left leg; θ_SYM-PSJT_: symmetry angle for the pivot step jump tests; SLJ: standing long jump.

**Table 7:** Intercorrelation matrix of the PSJT and the other jump tests for EGB (females, *n* = 20).

Test	CMJ r (*p*)	CMJA r (*p*)	CMJ_RLEG_ r (*p*)	CMJ_LLEG_ r (*p*)	SLJ r (*p*)	PSJ_RLEG_ r (*p*)	PSJ_LLEG_ r (*p*)
CMJ	-	0.94 * (<0.001)	0.92 * (<0.001)	0.94 * (<0.001)	0.68 * (0.031)	0.85 * (0.002)	0.83 * (0.003)
CMJA	0.94 * (<0.001)	-	0.79 * (<0.001)	0.88 * (<0.001)	0.64 * (0.003)	0.92 * (<0.001)	0.90 * (<0.001)
CMJ_RLEG_	0.92 * (<0.001)	0.79 * (<0.001)	-	0.80 * (<0.001)	0.48 * (0.033)	0.77 * (<0.001)	0.74 * (<0.001)
CMJ_LLEG_	0.94 * (<0.001)	0.88 * (<0.001)	0.80 * (<0.001)	-	0.53 * (0.016)	0.83 * (<0.001)	0.79 * (<0.001)
SLJ	0.68 * (0.031)	0.64 * (0.003)	0.48 * (0.033)	0.53 * (0.016)	-	0.71 * (<0.001)	0.81 * (<0.001)
PSJT_RLEG_	0.85 * (0.002)	0.92 * (<0.001)	0.77 * (<0.001)	0.83 * (<0.001)	0.71 * (<0.001)	-	0.93 * (<0.001)
PSJT_LLEG_	0.83 * (0.003)	0.90 * (<0.001)	0.74 * (<0.001)	0.79 * (<0.001)	0.81 * (<0.001)	0.93 * (<0.001)	-

* *p* < 0.05; CMJ: bilateral countermovement jump—arms akimbo; CMJA: bilateral countermovement jump with an arm swing; CMJ_RLEG_: unilateral countermovement jump performed with the right leg; CMJ_LLEG_: unilateral countermovement jump performed with the left leg; SLJ: standing long jump; PSJT_RLEG_: pivot step jump performed with the right leg; PSJT_LLEG_: pivot step jump performed with the left leg.

## Data Availability

The data presented in this study are available on reasonable request from the corresponding author.
